# Comparing postoperative complications of internal versus external fixation for closed diaphyseal tibial fractures in cats

**DOI:** 10.18849/ve.v11i2.737

**Published:** 2026-04-28

**Authors:** Stephen Keith John+, Jake Chitty+

**Keywords:** EXTERNAL FIXATION, FELINE, FRACTURE, INTERNAL FIXATION, ORTHOPAEDIC SURGERY, POSTOPERATIVE COMPLICATIONS

## Abstract

**PICO question:**

In cats with closed diaphyseal tibial fractures, does internal fixation, when compared to external fixation, result in a lower postoperative complication rate?

**Clinical bottom line:**

**Category of research:**

Treatment.

**Number and type of study designs reviewed:**

There were no publications that directly answered the PICO question.

**Strength of evidence:**

Zero.

**Outcomes reported:**

Both internal and external fixation are published techniques deployed in tibial fracture fixation in cats, however no study has directly compared the postoperative complications in these groups for closed diaphyseal tibial fractures.

**Conclusion:**

Given the lack of evidence to answer the PICO question, the veterinarian should consider their choice of stabilisation technique on the methods available and their personal experience. Both internal and external skeletal fixation (ESF) are published methods of feline tibial fracture fixation. A related study demonstrated that ESF has a high risk of complication for feline tibial fracture repair but given the absence of evidence for exclusively closed fracture types, this conclusion cannot be drawn for the PICO.

How to apply this evidence in practice

The application of evidence into practice should take into account multiple factors, not limited to: individual clinical expertise, patient’s circumstances and owners’ values, country, location or clinic where you work, the individual case in front of you, the availability of therapies and resources.

Knowledge Summaries are a resource to help reinforce or inform decision making. They do not override the responsibility or judgement of the practitioner to do what is best for the animal in their care.

## Clinical scenario

A 4-year-old domestic shorthair cat presents to your clinic with an acute non-weight bearing lameness of the right pelvic limb after falling from height. On clinical examination, you localise the pain and feel crepitus and instability at the level of the mid tibia. Radiographs reveal a closed mid-diaphyseal tibial fracture. The contralateral limb and pelvis are unaffected. Your clinic has the capability to perform external fixation and internal fixation for the repair, but you wish to deploy a technique that results in the lowest risk of developing postoperative complications.

## The evidence

The literature search found no evidence that addressed the PICO question from a literature search.

## Appraisal, application and reflection

Tibial fractures are relatively common in cats, accounting for approximately 10% of feline fractures (Hill, 1977), with 73% of these involving the diaphyseal region (Brunnberg et al, 2003). Tibial fracture fixation is understood to encounter higher rates of complications than fractures of other bones; non-union, delayed union, implant failure, and osteomyelitis being complications outlined in the literature (El-shafey et al., 2022; Brunnberg, 2003). Nolte et al. (2005) suggested that the over-presentation of non-union seen in tibial fractures could be associated with lack of soft tissue covering of the area. Other intrinsic factors such as a high cortical to cancellous bone ratio, lack of muscular attachments, and the biomechanical forces that act upon the tibia are other cited reasons for the higher rate of non-union seen (Glyde & Arentt, 2006). Extrinsic risk factors reported include age, weight, and degree of comminution (Nolte et al, 2005).

There are many techniques for open reduction and internal fixation (ORIF) including interlocking nail, bone plating, intramedullary pinning, and clamp-rod (DeCamp, 2006). These techniques can be applied in differing approaches including full open, Open But Do Not Touch (OBDNT), or minimally invasive plate osteosynthesis (MIPO) technique (Seaman & Simpson, 2004; Vannini, 2010; Schmierer & Pozzi, 2017; Butterworth, 2016; Duhautois, 2003). Advantages of MIPO included the minimal disruption to the biological environment, which may facilitate faster bone healing (Istim & Arican, 2022).

Internal fracture fixation is considered by veterinary surgeons as it facilitates accurate reduction and increased stability of fracture fragments to allow for bone healing (Pozzi et al., 2021). Unlike external fixation, implant removal for internal fixation is not anticipated and durability of the implants used is often increased, hence metalwork is less likely left in situ after explantation.

However, disadvantages of internal fixation include the possibility of increased tissue damage from the surgical approach potentially delaying healing, and internal implants serving as a nidus for infection (Johnston et al., 2018).  Therefore, the veterinary surgeon must consider the fracture patient assessment score and biological cost when planning fracture fixation methods, balanced against the mechanical and biological environment of the fracture.

External skeletal fixation (ESF) can be used in multiple different configurations such as uniplanar, biplanar, circular and circular-linear hybrid ESFs (Zurita & Craig, 2022) but generally consist of multiple pins with clamps and a connecting bar. Free form fixators have been described in literature, where epoxy or resin-acrylic bars can provide fixation, often at less expense than the other configurations mentioned previously (Roberts & Meeson, 2022). External fixation can be applied using closed reduction or via an OBDNT approach. External skeletal fixation stabilisation for fracture repair uses a biologic approach to osteosynthesis, allowing stability of the fractured bone and preservation of the haematoma and surrounding soft tissues (Palmer et al., 1992). However, the percutaneous nature of ESF means that infection is an increased risk compared to internal fixation (Chitty et al., 2025; Jaeger & Wosar, 2018).  A limitation to ESF is the anatomic reconstruction and fracture gap compression is challenging, if perfect anatomical reconstruction is not achieved a high strain environment can be created. This can be better addressed via anatomic reconstruction and rigid fixation, where more modern design plates such as Locking Compression Plates allow for compression of the fracture gap (Moreno et al., 2018; Haaland et al., 2009).

There was no evidence that directly addressed the PICO question, although one published study (Perry & Bruce, 2015) compared complications between internal fixation via ORIF to ESF in tibial fractures in 57 cats aged between 3 months and 200 months. Perry & Bruce (2015) defined complications as ‘any undesirable outcome associated with the surgical procedure’ and further classified major complications where surgical intervention was required, and minor where the complication could be conservatively managed. Of the total complication rate of 23/59 (40.4%) in the study, only one of these was in the ORIF group. Conversely, 50% (22/44) of all ESF fixations encountered at least a minor complication. Complications included pin tract infection/pin loosening, delayed union, non-union, pin loosening in absence of any infection, osteomyelitis, frame disruption leading to fragment displacement, and mild distal tibial valgus deformity. All nine major complications were encountered in the ESF group; four of these cases required limb amputation, another four cases required surgical revision, and one case was euthanised due to extreme lethargy of unknown aetiology. When specifically assessing infection, the ORIF group had no reported postoperative infections, whereas the ESF group had a postoperative infection rate of 27.2% (12/44). This study included 16/59 cats (27%) with open fractures within their evaluation. Direct comparison of this study to the PICO should be acknowledged cautiously as open fractures were included which may have influenced the overall postoperative complication rate. This study was a retrospective study with low case numbers which sits low on the hierarchy of evidence (Howick et al., 2011).

A study by Beever et al. (2017) analysed postoperative complications when ESFs were deployed for feline fracture stabilisation. Of the 140 cats included in the study, 24% (34/140) of these had tibial fractures. This study found that only 3% (1/34) of tibial fractures stabilised by ESF produced fixator-associated complications, superficial pin tract infection being the only reported postoperative complication. Limitations of this study include its retrospective nature with a small number of cases, so it sits low on the hierarchy of evidence.

Considering the absence of evidence directly addressing the PICO question, veterinary surgeons should deploy the techniques that they are competent in and are comfortable using from personal experience of fracture stabilisation and repair, with consideration of the patient and owners’ limitations/ability to manage postoperative care.

A prospective or retrospective study comparing postoperative complications with internal versus external fixation for closed diaphyseal tibial fractures in cats is required for the PICO to be addressed.

In conclusion, there is no evidence that directly compared internal fixation to external fixation for cats with closed tibial fractures. Related literature shows that internal fixation does carry a lower complication rate when compared to ESF when all types of fractures are considered, but no conclusion can be drawn from the available evidence on whether this would be applicable to exclusively closed fracture types.

## Methodology

**Search Strategy:** 

Databases searched and dates covered:	CAB Abstracts on OVID interface 1973 to 2025 Week 07PubMed Accessed via the NCBI website 1920 to February 2025
Search strategy:	CAB Abstracts: (cat or cats or feline or felines).mp. or exp cats/ ((tibia or tibial).mp. or exp tibia/) and ((fracture or fractures).mp. or exp fracture/) ((fixation or fixator or stabilise or stabilize) and (internal or external)).mp. exp fracture fixation/ 1 and 2 and (3 or 4) PubMed: cat OR feline (tibia OR tibial) AND fracture (fixation OR fixator OR stabilise OR stabilize) AND (internal OR external) 4. 1 AND 2 AND 3
Dates searches performed:	21 February 2025

**Exclusion / Inclusion Criteria:** 

Exclusion:	Studies including open fractures Only on technique deployed Articles irrelevant to PICO Book chapters Case reports
Inclusion:	Prospective studies Retrospective studies

**Search Outcome:** 

Database	Number of results	Excluded – not specific to PICO question	Total relevant papers
CAB Abstracts	160	160	0
PubMed	71	71	0
Total relevant papers when duplicates removed			0

## Author contributions

**Stephen Keith John:** Writing - Original Draft, Writing - Review & Editing. **Jake Chitty:** Writing - Review & Editing

## ORCiD

Stephen Keith John: https://orcid.org/0009-0003-2581-043X Jake Chitty: https://orcid.org/0000-0001-7011-006X

## Conflict of Interest

The authors declare no conflicts of interest.

## Disclaimer

Knowledge Summaries are a peer-reviewed article type which aims to answer a clinical question based on the best available current evidence. It does not override the responsibility of the practitioner. Informed decisions should be made by considering such factors as individual clinical expertise and judgement along with patient’s circumstances and owners’ values. Knowledge Summaries are a resource to help inform and any opinions expressed within the Knowledge Summaries are the author's own and do not necessarily reflect the view of the RCVS Knowledge. Authors are responsible for the accuracy of the content. While the Editor and Publisher believe that all content herein are in accord with current recommendations and practice at the time of publication, they accept no legal responsibility for any errors or omissions, and make no warranty, express or implied, with respect to material contained within. For further information please refer to our Terms of Use.
